# Case Report: Reversible cabergoline-associated cardiac valvulopathy post drug discontinuation

**DOI:** 10.12688/f1000research.3062.1

**Published:** 2014-07-25

**Authors:** Chris G. Yedinak, Shirley McCartney, Troy H. Dillard, Kevin S. Wei, Maria Fleseriu

**Affiliations:** 1 Northwest Pituitary Center, Oregon Health & Science University, Portland, Oregon, 97239, USA; 2 Department of Neurological Surgery, Oregon Health & Science University, Portland, Oregon, 97239, USA; 3 Department of Medicine, Division of Endocrinology, Diabetes and Clinical Nutrition, Oregon Health & Science University, Portland, Oregon, 97239, USA; 4 Department of Cardiovascular Medicine, Oregon Health & Science University, Portland, Oregon, 97239, USA

## Abstract

We present a case of a 21 year old male patient diagnosed with a 2.2 cm prolactin-secreting adenoma in contact with the optic chiasm. The patient was treated with up to 6mg/week of cabergoline (total cumulative dose 814 mg) and developed mild valvulopathy. Valvulopathy was subsequently reversed after discontinuation of cabergoline therapy.

## Presentation

A 21 year old male patient with a history of delayed puberty and a 2.2 cm pituitary adenoma was referred for evaluation. At presentation, prolactin level was found to be greater than 1000 ng/ml (normal range: 3–13 ng/ml) on diluted testing. Treatment with the dopamine agonist (DA) cabergoline was initiated and over a 10-month period, the macroadenoma shrank substantially to < 1cm (as observed by magnetic resonance imaging). Total cabergoline dose at that time was 4.5 mg/week, in three equally divided weekly doses. Neither prolactin level nor testosterone level normalized. The patient reported no symptoms as would have been expected of a prolactinoma of this size; he denied visual field changes or deficits, galactorrhea, breast discomfort, fatigue, temperature dysregulation, weight changes or fluctuations, hair loss, weakness, memory changes or muscle loss. He felt his libido was somewhat diminished and morning erections were absent. He did report headaches at presentation that resolved after 10 months of treatment with cabergoline.

Medical history was significant only for reactive airway disease for which he was treated with albuterol (as needed), which was seldom used. He was not sexually active, was a non-smoker with infrequent alcohol use and no caffeine intake. The patient confirmed a family history of cancer and arthritis in a grandmother and hypertension in a grandfather, but a negative family history of cardiac disorders, pituitary masses or known heritable diseases.

On physical exam he was normotensive 119/82 mmHg, pulse 81, weight 90.7 Kg, height 1.78 m (body surface area 2.06 m ^2^). He did not have visual field deficit to confrontation and had a normal cardiac exam without murmur, rub or gallop. Chest was clear to auscultation and breast exam revealed no gynecomastia, tenderness or nipple discharge. He was void of body hair on his chin, chest, arms and legs. The genitourinary exam revealed pre-pubertal testes with no pubic hair and small penile size. The remainder of his physical exam was unremarkable.

**Figure 1.**
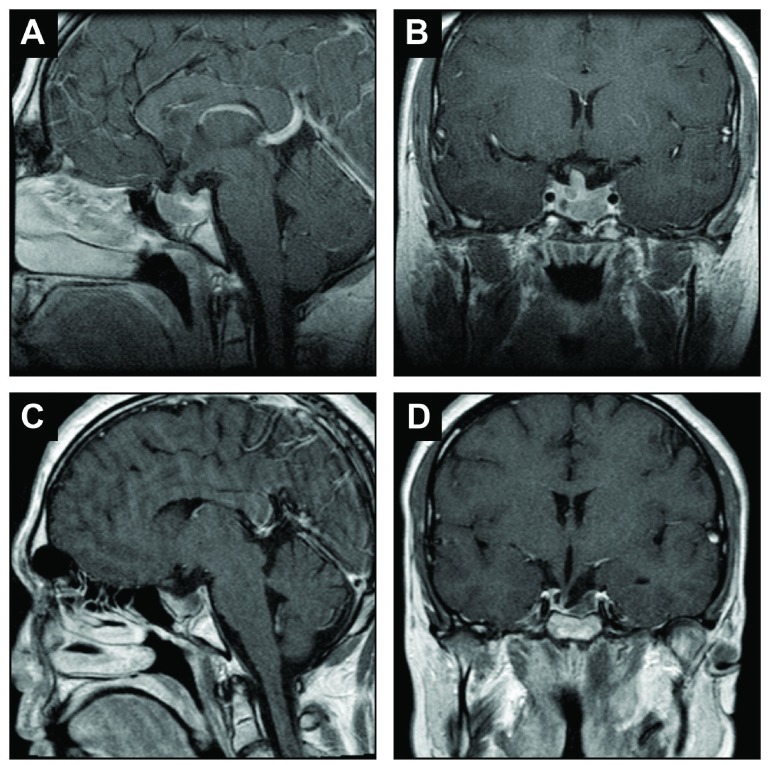
Magnetic resonance imaging: pre-treatment ( **A**) sagittal T1 and ( **B**) coronal T1, and 2 years post-treatment ( **C**) sagittal T1 and ( **D**) coronal T1.

Laboratory evaluation indicated a testosterone level 51 ng/dL (normal range: 241–950 ng/dL) with follicle-stimulating hormone (FSH) and luteinizing hormone (LH) of 1 mlU/mL (inappropriately low; normal adult male range FSH 1.5–12.4 mIU/ml and LH 2.1–4.7 mIU/ml). Thyroid axis was noted to be intact with thyroid stimulating hormone of 2.1 µlU/ml (normal range: 0.34–5.6 µlU/ml) and free thyroxine (T4) of 0.7 ng/dL (0.6–1.2 ng/dL). Prolactin level was 55 ng/ml (normal range: 3–13 ng/ml). A cosyntropin stimulation test was performed to evaluate the hypothalamic-pituitary-adrenal (HPA) axis function with a normal baseline cortisol level at 9:20 am of 14.3 µg/dL and adrenocorticotropin hormone (ACTH) of 41 pg/mL (< 46 pg/ml). His cortisol level was minimally blunted at 17.2 µg/dL (normal range: > 18 µg/dL) post stimulation with low dose (1 μg) Cortrosyn. IGF-1 was low at 84 ng/mL (age and gender adjusted normal 116–358 ng/mL). Magnetic resonance (MR) imaging prior to cabergoline treatment, indicated a 2.2 cm tumor in contact with optic chiasm ( Figure 1A, B); follow up MR imaging after 10 months of cabergoline treatment revealed residual tumor on the left side of the gland (< 1cm) without optic chiasm involvement ( Figure 1C, D). The patient’s bone age was evaluated as 16.5 years.

## Diagnosis

The presence of a pituitary macroadenoma, hypogonadism and a single measurement of serum prolactin > 250 ng/mL confirmed a diagnosis of prolactinoma ^1^. Likewise, drug-induced hyperprolactinemia, elevation related to stalk effect and drug effects were excluded by this degree of elevation.

There was no evidence of co-secretion with growth hormone and the primary diagnosis remained hyperprolactinemia related to a prolactin-secreting pituitary adenoma with central hypogonadotrophic hypogonadism. Given the significant tumor shrinkage, DA therapy with cabergoline was continued without dose change. No testosterone replacement was initiated, in anticipation of further tumor response to DA therapy and subsequently normalization of both the prolactin level and spontaneous puberty. However, low bone age raised concern for growth hormone deficiency versus hypogonadism from possible pituitary damage and low dose depot testosterone (50 mg intramuscular monthly) and growth hormone replacement (0.2 mg/daily) were started for a trial of 6 months. As prolactin level remained elevated after a further 3 months of treatment, cabergoline dose was increased to 5 mg in a divided dose twice a week. He continued to tolerate DA therapy without side effects.

At follow up testing, after an additional 3 months of treatment, HPA axis function had not improved and he was treated with low dose glucocorticoids (hydrocortisone 10 mg daily) and thyroid (levothyroxine 50 mcg daily) replacement. After a further 12 months of treatment, prolactin level was reduced to 22 ng/ml (normal range: 3–17 ng/ml), but did not normalize; however tumor remained stable. Cabergoline dose was increased to a total of 6 mg weekly in three divided doses. After a total of 2 years of treatment with both DA and testosterone replacement, he began to gain muscle mass and achieved Tanner 4/5 with some penile growth.

**Figure 2.**
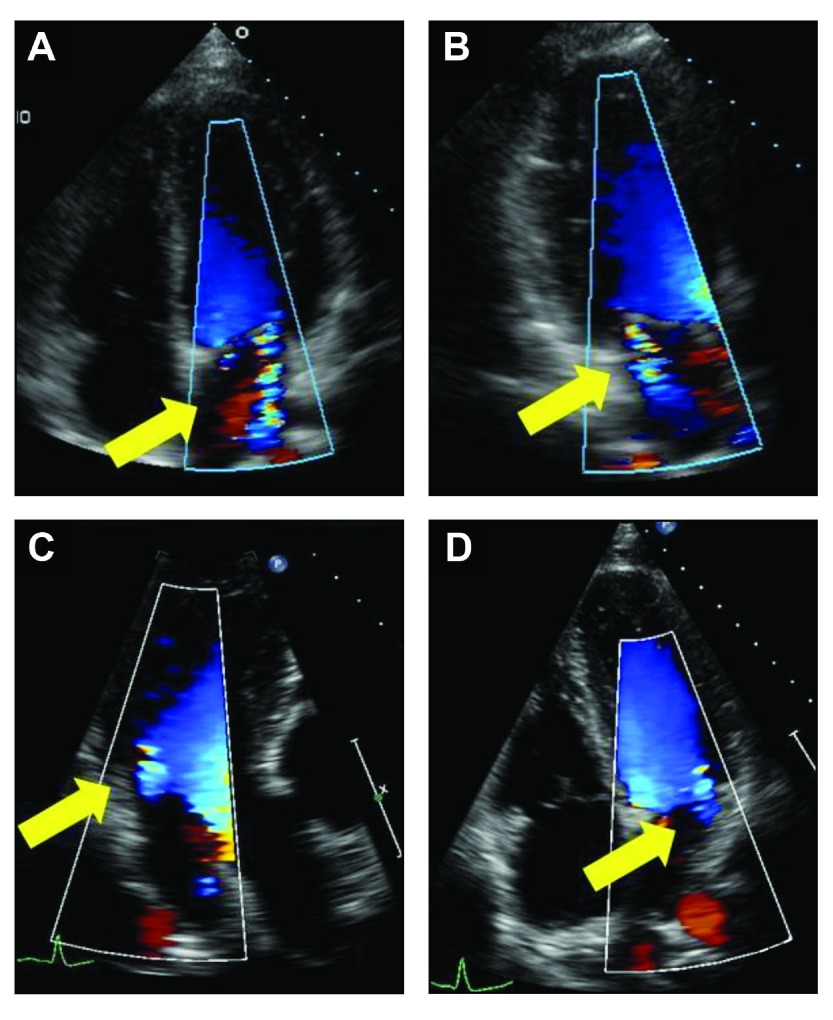
Echocardiograms: baseline ( **A** and **B**) demonstrates apical displacement during systole with mild non-coaptation of the mitral leaflets and mild mitral regurgitation (arrows). Follow-up ( **C** and **D**), 8 months after discontinuing cabergoline and starting bromocriptine, demonstrates a normal valve without mitral regurgitation (arrows).

After an accumulative dose of 814 mg cabergoline over 4 years of treatment, the patient was found to have developed a faint systolic murmur on auscultation. A 2D, color and spectral Doppler echocardiogram indicated mild non-coaptation of the mitral leaflets associated with slight apically displacement in systole, normal valve thickness and excursion resulting in mild mitral regurgitation ( Figure 2A, B). Ejection fraction was estimated at 60–65% with normal left ventricular function. Doppler measures of mitral valve peak E 1.08 m/s and peak A 0.29 m/s with E/A ratio 3.75 where E (early wave) represents passive filling of the ventricle and A (atrial wave) the active filling with atrial systole. Classically, the E-wave velocity is slightly greater than that of the A wave. All other values demonstrated normal structure and function. Changes were reported to be consistent with cabergoline therapy. Cabergoline was subsequently discontinued and DA therapy was continued using bromocriptine 5 mg/daily. The patient’s prolactin level increased to 70 ng/ml (normal range: 3–13 ng/ml) 2 months after switching from cabergoline therapy to bromocriptine.

MR imaging performed after 6 months of treatment with bromocriptine was unchanged. All other pituitary hormonal levels were stable and no other concomitant medications had been used. Repeat echocardiography (performed in the same modes by the same sonographer and reviewed by the same cardiologist), revealed normal mitral valve function with Doppler measures of mitral valve peak E of 0.68 m/s and peak A 0.32 m/s and E/A ratio 2.14 but moderately reduced global RV systolic function and LV function of 55–60% ( Figure 2C, D). Clinical exam, including vital signs, was unchanged between these two visits, the bromocriptine dose was increased to 10 mg daily and the patient was referred for cardiac consultation.

At cardiac consultation (3 months post bromocriptine dose increase) the patient did not have a murmur on exam. On review of the echocardiogram no appreciable evidence was found for ongoing disease and follow up echocardiography was recommended after a further 6 months. At this exam, a normal LV systolic function was found with an LV ejection fraction estimated at 62% using biplane Simpson’s method. No other echocardiographic abnormalities were found and improvement from previous echocardiograms was reported.

## Discussion

Cabergoline has been implicated in the induction of fibrotic cardiac valvulopathy when used in high doses mostly for the treatment of Parkinson’s disease ^2, 3^ but also in treatment of prolactinomas ^4– 6^. Other studies have not shown a significant increase in cardiac valvular disease when cabergoline was used in patients with pituitary diseases ^7– 10^. The pathophysiologic mechanism of DA-related valvular heart disease is thought to be related to interactions of the drug with serotonin (5-HT) receptors, particularly 5-HT _2B_ receptors. This receptor is present in fibroblasts on heart valves in high concentrations, but is also present in pulmonary arteries ^11– 13^.

Two independent researchers in large European studies ^14, 15^ reported an association between high doses of DAs (associated with the treatment of Parkinson’s disease) with potent 5-HT _2B_ agonist activity and cardiac valve disease: particularly of the mitral, aortic and tricuspid valves. Activation of these receptors has been demonstrated to lead to excess cell division and overgrowth valvulopathy and dysfunction ^16^. Only pergolide ^16^ and cabergoline demonstrate 5-HT _2B_ receptor activity. In a study by Zanettini *et al.* rates of drug induced valvulopathy occurred in 28.6% of treated patients ^14^.

Bromocriptine was thought to be devoid of this activity ^15^, but partial 5-HT _2B_ receptor activity was subsequently demonstrated in porcine models ^17^. However, studies of this effect for bromocriptine in patients treated for prolactinomas are scarce. Two studies of subjects treated long term (mean 54.8 vs 58.98 months) are available: 55 subjects (58.98 months) that reported significantly higher end diastolic intraventricular septal thickness with bromocriptine ^5^; and 19 cases (54.8 months) with a higher prevalence of tricuspid regurgitation. Several case reports of bromocriptine use for Parkinson’s disease reported a higher incidence of pleuropericarditis in association with long term use ^5, 18^.

## Management

Although there are reports of reversibility of valvulopathy with other DAs ^19^, this is the first case of reversal of valvular abnormalities after stopping cabergoline treatment in a patient with a prolactin-secreting adenoma. It is unclear if the risk of cardiac valvulopathy is associated with a high cumulative dose ^19^ or is gender related ^20^. However, this case highlights the potential reversibility of mild valvulopathy associated with cabergoline therapy if treatment is discontinued prior to the onset of severe structural abnormalities.

Reversibility of ergot-derived DA and 5HT _2B_ agonist-induced valvular heart disease is infrequently documented and mostly limited to patients with Parkinson’s disease or weight loss treatment. In a rodent model, 12 weeks of serotonin injections induced both aortic and mitral regurgitation. Eight weeks after cessation of serotonin injections, the prevalence of valvulopathy was no longer higher than control and valvular thickness returned to baseline ^19^. In 50 patients previously given fenfluramine or dexfenfluramine, valvular regurgitation in 17 of the 38 (45%) patients with mitral regurgitation and 19 of the 43 (44%) patients with aortic regurgitation improved after stopping the drug ^12^. Regression of mitral valve disease was also noted in 4/10 patients who had discontinued pergolide (for Parkinson’s) in the previous 4–6 months ^21^. To the best of our knowledge, only one case (1 of 4 treated for Parkinson’s disease) of reversible mitral valve disease related to cabergoline discontinuation has been described ^2^.

The potential for cardiac valve effects in patients treated for prolactinomas has been previously reported ^3, 4, 22^, but subsequent studies have not confirmed these findings ^7, 9, 23^. Cabergoline remains an effective treatment to normalize prolactin levels and for tumor shrinkage in patients with prolactinomas ^1^. Management recommendations include using the lowest dose for the shortest period possible to achieve these objectives, while monitoring for the development of flow murmurs at each visit. Echocardiographic evaluation should be considered in patients who require long-term treatment with cabergoline, especially in high doses. Guidelines for repeat echocardiographic intervals remain unclear. There is a need for larger, preferably prospective, studies with careful echocardiographic assessment and with longer durations of follow-up than the currently available studies.

Should valvular regurgitation develop, early discontinuation and management with bromocriptine may be effective in reversing cardiac valvular dysfunction, as observed in this case study.

## Consent

Written informed consent for publication of clinical details and clinical images was obtained from the patient. The OHSU institutional review board does not require additional consent for case reports.
