# Supplementary material for: Case Report: Reversible cabergoline-associated cardiac valvulopathy post drug discontinuation
Source: F1000Res. 2014 Jul 25;3:171. [Version 1] doi: 10.12688/f1000research.3062.1 (PMC4133764; doi:10.12688/f1000research.3062.1)
Supplement: Peer review history

# Peer review: Referee response for version 1

**DOI:** 10.5256/f1000research.3298.r5593

**Published:** 2014-08-11

**Authors:** Corin Badiu

**Affiliations:** 1 Department of Endocrinology, “Carol Davila” University of Medicine and Pharmacy, Bucharest, Romania

The authors present a case of prolactinoma in a young man. The case is instructive in terms of cabergoline impact upon associated valvulopathy.

Few changes are required in order to improve the presentation / educational outcomes of the manuscript.

The case is a partial resistant prolactinoma, since prolactin values remained above normal level and required a high CAB dosage after 10 months of treatment. The clinical examination should specify Tanner pubertal stage at initial presentation, since it is mentioned after 2 years of treatment. What was the effect of this treatment on bone age (previously mentioned as delayed)? No data are presented about fertility evaluation / treatment. For a man evaluated between 21-25 years, this might be of importance.

Concerning heart evaluation, presented at 4 years after beginning of treatment, the authors should mention if they did heart ultrasound ever before? Some subclinical aspects can be revealed by detailed regular heart evaluation, especially on patients on high doses of Cabergoline.

Obviously, alternative treatments should be commented on. First and foremost surgical approach, when the tumor became microadenoma, is a very reasonable way to treat without significant side-effects. The authors should comment on this option.

I have read this submission. I believe that I have an appropriate level of expertise to confirm that it is of an acceptable scientific standard, however I have significant reservations, as outlined above.

# Peer review: Referee response for version 1

**DOI:** 10.5256/f1000research.3298.r5591

**Published:** 2014-08-06

**Authors:** Niki Karavitaki

**Affiliations:** 1 Department of Endocrinology, Oxford Centre for Diabetes, Endocrinology and Metabolism, Oxford, UK

Very interesting case.

I have read this submission. I believe that I have an appropriate level of expertise to confirm that it is of an acceptable scientific standard.

# Peer review: Referee response for version 1

**DOI:** 10.5256/f1000research.3298.r5594

**Published:** 2014-08-04

**Authors:** Atanaska Elenkova

**Affiliations:** 1 Department of Hypothalamic, Pituitary, Adrenal and Gonadal Diseases, Clinical Centre of Endocrinology, Sofia, Bulgaria

The article is well written, treats an actual problem (the risk of development of valvulopathy after long-term cabergoline treatment in patients with macroprolactinoma) and provides evidence about the reversibility of valvular changes after timely discontinuation of DA treatment.

**Title and abstract: **The title is appropriate for the content of the article. The abstract is concise and accurately summarizes the essential information of the paper although it would be better if the authors define more precisely the anatomic specificity of valvulopathy – mild mitral regurgitation.

**Case report:** The clinical case presentation is comprehensive and detailed but there are some minor points that should be clarified:

1. Please clarify the prolactin levels at diagnosis. In the *Presentation *section (line 3) “At presentation, prolactin level was found to be grater than 1000 ng/ml on diluted testing” but in the section describing the laboratory evaluation at diagnosis (line 7) “Prolactin level was 55 ng/ml”. Was the difference due to so called “hook effect”?  
2. Figure 1: In the text the follow-up MR imaging is indicated to be “ *after 10 months of cabergoline treatment*”. However, the figures 1C and 1D represent 2 years post-treatment MR images. Please clarify.
3. Figure 2: Echocardiograms 2A and 2B are defined as baseline but actually they correspond to the follow-up echocardiographic assessment at the 4 ^th^ year of cabergoline treatment. Did the patient undergo a baseline (prior to dopamine agonist treatment) echocardiographic evaluation? If he did not, it should be mentioned as study limitation in the *Discussion *section.
4. The mitral valve thickness was mentioned to be normal. Did the echographic examination visualize increased echogenicity (hyperechogenicity) of the mitral cusps?
    
5. How could you explain the decrease of LV ejection fraction (from 60-65% to 50-55%) after switching from cabergoline to bromocriptine treatment and respectively its increase to 62% after doubling the bromocriptine daily dose? Was LV function estimated always by the same method during the follow-up?
6. **Final paragraph:** Authors conclude that early discontinuation and management with bromocriptine may be effective in reversing cardiac valvular dysfunction. Even though, regular echocardiographic follow up should be considered in patients who are expected to be on long-term high dose treatment with bromocriptine regarding its partial 5-HT2b agonist activity.

I have read this submission. I believe that I have an appropriate level of expertise to confirm that it is of an acceptable scientific standard.
